# Exposure to Blue Wavelength Light Is Associated With Increases in Bidirectional Amygdala-DLPFC Connectivity at Rest

**DOI:** 10.3389/fneur.2021.625443

**Published:** 2021-03-26

**Authors:** Anna Alkozei, Natalie S. Dailey, Sahil Bajaj, John R. Vanuk, Adam C. Raikes, William D. S. Killgore

**Affiliations:** ^1^Social, Cognitive, and Affective Neuroscience Laboratory, Department of Psychiatry, University of Arizona, Tucson, AZ, United States; ^2^Multimodal Clinical Neuroimaging Laboratory (MCNL), Center for Neurobehavioral Research, Boys Town National Research Hospital, Boys Town, NE, United States

**Keywords:** fMRI, depression, light therapy, neuroimaging, amygdala, PFC

## Abstract

Blue wavelength light has been used successfully as a treatment method for certain mood disorders, but, the underlying mechanisms behind the mood enhancing effects of light remain poorly understood. We investigated the effects of a single dose of 30 min of blue wavelength light (*n* = 17) vs. amber wavelength light (*n* = 12) exposure in a sample of healthy adults on subsequent resting-state functional and directed connectivity, and associations with changes in state affect. Individuals who received blue vs. amber wavelength light showed greater positive connectivity between the right amygdala and a region within the left dorsolateral prefrontal cortex (DLPFC). In addition, using granger causality, the findings showed that individuals who received blue wavelength light displayed greater bidirectional information flow between these two regions relative to amber light. Furthermore, the strength of amygdala-DLPFC functional connectivity was associated with greater decreases in negative mood for the blue, but not the amber light condition. Blue light exposure may positively influence mood by modulating greater information flow between the amygdala and the DLPFC, which may result in greater engagement of cognitive control strategies that are needed to perceive and regulate arousal and mood.

## Introduction

Light has a powerful effect on mood. A sunny day often seems to enhance positive outlook and helping behavior (1, 2), and it is well-established that bright light can be an effective treatment for mood disorders, with effects as potent as some pharmacologic treatments (3, 4). Even a single half-hour exposure to bright light seems to improve mood (5). Moreover, the wavelength of light also seems to be important to these effects, with considerable evidence now pointing to the importance of blue wavelengths (460–480 nm) on mood and cognition. Specifically, studies have shown that acute exposure to blue wavelength or bright broad-spectrum light leads to immediate increases in simple alertness and attention (6, 7) as well as more complex cognitive functions, such as improved working memory performance (8) and short-term verbal memory retention (9). Daily morning blue wavelength light exposure over several weeks has also been used as an effective treatment for seasonal and non-seasonal depression (10–13), certain sleep disorders, such as delayed sleep phase syndrome (14), and to improve symptoms of fatigue in individuals with neurological conditions, such as acquired brain injury (15–18). The exact mechanisms underlying these beneficial effects of blue light on emotion and cognition, however, are not well-understood.

Growing evidence suggests that many of these mood and cognitive effects of light are likely mediated by the non-image forming pathways of the visual system. It is well-documented that bright light, especially within the blue wavelengths, stimulates intrinsically photosensitive retinal ganglion cells (ipRGCs) which transmit signals to several sub-cortical nuclei. These include the suprachiasmatic nucleus (SCN) in the hypothalamus, which has a strong influence on the circadian rhythm of sleep and wake (19, 20). Extensive research has shown that exposure to bright light, particularly in the blue wavelengths, leads to activation of the SCN, which in turn sends signals to the pineal gland to suppress the production of the hormone melatonin (21, 22). Melatonin is released and suppressed daily in accordance with the circadian rhythm, and its release precedes sleep onset (23). Consequently, exposure to blue light at times that are out of phase with the normal circadian rhythm can lead to disruptions in the sleep-wake schedule. For instance, blue light exposure during the night, when melatonin levels are high, appears to lead to increases in alertness by suppressing the immediate production of melatonin (6) and phase delaying the circadian onset of sleep. Conversely, light in the morning hours will produce a phase advance in the circadian rhythm. Notably, by targeting light exposure to specific times in the day, the rhythm of melatonin release can be shifted to treat certain sleep disorders (e.g., to treat delayed sleep phase syndrome, daily morning bright light exposure over several days leads to a phase advance of melatonin release and earlier sleep onset) (24). It has been proposed that seasonal depression is associated with a disturbance in the circadian rhythm during the darker winter months, and that among affected individuals, daily bright light treatment may produce its mood-enhancing effect, in part, by “resynchronizing” the biological clock (25).

While circadian factors have been suggested for the outcomes mentioned above, they do not appear to account for all of the mood and cognitive enhancing effects of blue light. For example, suppression of melatonin cannot effectively account for the alerting effects of light during the middle of the day, when melatonin levels are already naturally low, or for the effects of light on non-seasonal depression (i.e., during times of year when sunlight is more prevalent). Direct projections from the ipRGCs, as well as indirect projections from the SCN, to other brain regions involved in emotion and cognition, are proposed to explain some of those non-circadian effects (26).

For example, there is evidence from several animal studies to suggest that ipRGCs have direct projections to the medial amygdala (27–30) which may therefore also be modulated by blue light exposure. In fact, studies in human subjects have shown that exposure to blue vs. green light in the MRI scanner led to immediate increases in activity within the right amygdala that declined over time, suggesting a habituation process (31, 32), and enhanced connectivity between the left amygdala and the voice sensitive area of the superior temporal gyrus and the hypothalamus during an auditory emotional processing task during blue vs. green light (32). Further, one study in healthy males demonstrated that 3 weeks of bright light therapy reduced bilateral amygdala and prefrontal reactivity to threat (i.e., angry and fearful faces) in a dose-dependent manner; and that left amygdala and medial prefrontal connectivity also increased in a dose-dependent manner (33). The role of blue light exposure in modulating amygdala activation potentially as a result of direct projections from ipRGCs may be particularly relevant for individuals with mood disorders, such as depression. Compared to healthy controls, individuals with depression show increased amygdala reactivity to negative stimuli, and disrupted resting-state functional connectivity, such as hypoconnectivity between the amygdala and several areas of the prefrontal cortex, which may underlie difficulties with emotion regulation (34–36).

Considering that blue wavelength light appears to lead to immediate increases in activation in the right amygdala (31), and that 3 weeks of bright light therapy led to increases in connectivity between the amygdala and PFC (33), it is possible that exposure to blue wavelength light may also lead to more *immediate* changes in functional connectivity between the amygdala and PFC. Because negative mood states are often associated with reduced prefrontal regulation and increased amygdala activation, alterations in the strength of connectivity via light exposure may contribute to its well-established antidepressant or mood-enhancing effects.

The aim of this study was to answer these research questions by investigating the effects of a single, 30-min controlled exposure to blue vs. non-blue (amber) light on subsequent (i) positive and negative affect, (ii) functional and directed brain connectivity of the amygdala as a seed with other brain regions, and (iii) the association between changes in affect and observed changes in functional brain connectivity.

Considering the scarcity of previous findings of the effects of blue wavelength light on amygdala responsiveness and connectivity, we have deliberately kept our hypotheses broad (e.g., have not made specific hypotheses regarding laterality or connectivity patterns). The following hypotheses were postulated:

1. Blue light exposure would increase positive affect and decrease negative affect in comparison to amber light exposure.
2. Blue light exposure would lead to increased functional and directed functional connectivity between the amygdala and the PFC.
3. Connectivity patterns would be associated with more positive and less negative affect.

## Methods

### Participants

Twenty-nine healthy adults between 18 and 32 years of age (M = 21.52, SD = 2.82; 16 men, 13 women) took part in the study and provided useable functional and structural magnetic resonance imaging (MRI) data for analysis (out of a total of 35 participants). Six participants had to be excluded due to excessive head movement in the scanner. Participants completed an average of 14.1 years of education (SD = 1.95). According to self-report, all were right handed, primary English speaking, and reported a regular sleep schedule of going to bed between 10:00 p.m. and 1:00 a.m. and waking between 6:00 and 9:00 a.m. Further, participants were screened for a history of major psychiatric disorders, including major depressive disorder, anxiety disorders, obsessive-compulsive disorders, post-traumatic stress disorder and substance use disorders. In addition, participants were asked whether they had ever been in psychiatric treatment and for what reason. Participants were asked to keep their regular sleep schedule and were asked to consume their normal levels of caffeine on the day of the study. Participants were randomly assigned to the blue (*n* = 17) or amber light condition (*n* = 12) (see below). The two groups did not differ on age, sex, number of hours slept the night before the assessment, or number of reported hours slept on weeknights (see Table 1). In addition, four participants in the blue light group and four participants in the amber light group reported having had one caffeinated product on the day of the assessment. Separate data from this same study have been reported elsewhere (8, 9, 37, 38), but the functional connectivity and mood findings reported here are novel and have not been previously published. This project was approved by the Institutional Review Board at the University of Arizona and the U.S. Army Human Research Protections Office, and all participants provided written informed consent prior to study participation.

**Table 1:** Descriptive statistics.

	**Blue group (*n* = 17) Mean (SD)**	**Amber group (*n* = 12) Mean (SD)**	**Statistic**
Age	21.47 (2.85)	21.58 (2.91)	*t*_(27)_ = −0.10. *p* = 0.92
Sex	47.05% female	41.66% female	χ^2^ = 0.08, *p* = 0.77
Number of hours slept on weeknights	7.25 (0.97)	7.29 (1.015)	*t*_(27)_ = −0.11, *p* = 0.91
Number of hours slept the night prior to the assessment	6.88 (0.54)	6.79 (.62)	*t*_(27)_ = 0.42, *p* = 0.68
BDI-II	1.53 (1.94)	2.58 (3.32)	*t*_(27)_ = −1.08, *p* = 0.29
PANAS-P pre-light	28.94 (8.97)	28.00 (5.46)	*t*_(27)_ = 0.32, *p* = 0.75
PANAS-P post-light	27.18 (11.40)	24.25 (8.35)	*t*_(27)_ = 0.75, *p* = 0.45
PANAS-N pre-light	12.29 (1.44)	12.67 (2.46)	*t*_(27)_ = −0.51, *p* = 0.61
PANAS-N post-light	11.00 (2.42)	10.42 (.80)	*t*_(27)_ = 0.80, *p* = 0.43

*BDI-II, Beck Depression Inventory; PANAS, Positive and Negative Affective Schedule (PANAS-P: positive affect, PANAS-N: negative affect)*.

### Materials

#### Positive and Negative Affect Schedule (PANAS)

The PANAS (39) was used to measure positive and negative affect. Participants were asked to indicate, on a 5-point scale, the extent to which (from “very slightly to not at all” to “extremely”) they were feeling a number of positive (e.g., interested, excited, proud) and negative feelings (e.g., nervous, determined, irritable) at that very moment. Total scores for positive affect (PANAS-P) and negative affect (PANAS-N) were calculated separately and each had a possible range from 10 to 50. The PANAS is a widely used measure of state affect that has shown good internal consistency, reliability, and validity (40).

#### Light Exposure

The light protocol is described in detail in the Procedure section. The blue light exposure devices were commercially available Philips goLITE BLU® Energy Light devices (Model HF3321/60; Philips Electronics, Stamford, CT). The amber light devices were provided by the manufacturer for research purposes and were virtually identical to the goLITE BLU devices, with the exception of being fit with a different color LED. Each device consisted of a plastic table-mounted chassis with a 10 × 6 array of light emitting diodes (LEDs), encased in 1 × 1 cm cubical projection elements and a translucent plastic window cover. The light devices were set up in such a way that they were centered at 45° to each side of the participant with a distance of ~80 cm from the participant's nasion which means that only part of the field of view was covered. The goLITE BLU Energy Light is commercially available and has a narrow bandwidth [peaking at λ = 469 nm, at 214 Lux at eye level, and panel irradiance (mW/cm^2^) = 1.23]. The amber devices were otherwise identical to the goLITE BLU devices, with the exception that they were fitted with amber LEDs [peaking at λ = 578 nm, at 188 Lux at eye level, and total irradiance (mW/cm^2^) = 0.35]. The alpha-opic irradiances of the blue and amber conditions are summarized in Table 2.

**Table 2:** Alpha-opic irradiances of the light conditions.

	**S-cone-opic**	**M-cone-opic**	**L-cone-opic**	**Rhodopic**	**Melanopic**
**BLUE WAVELENGTH LIGHT**					
α-opic irradiance, W·m^−2^	1.36	0.77	0.46	1.74	2.09
α-opic efficacy of luminous radiation, mW·lm^−1^	6.30	3.57	2.13	8.09	9.69
α-opic equivalent daylight (D65) illuminance, lx	1660.04	528.75	282.19	1202.59	1573.80
**AMBER WAVELENGTH LIGHT**					
α-opic irradiance, W·m^−2^	0.01	0.20	0.29	0.05	0.02
α-opic efficacy of luminous radiation, mW·lm^−1^	0.03	1.10	1.59	0.30	0.11
α-opic equivalent daylight (D65) illuminance, lx	7.27	136.11	175.24	36.90	14.53

### Procedure

**Figure 1:**
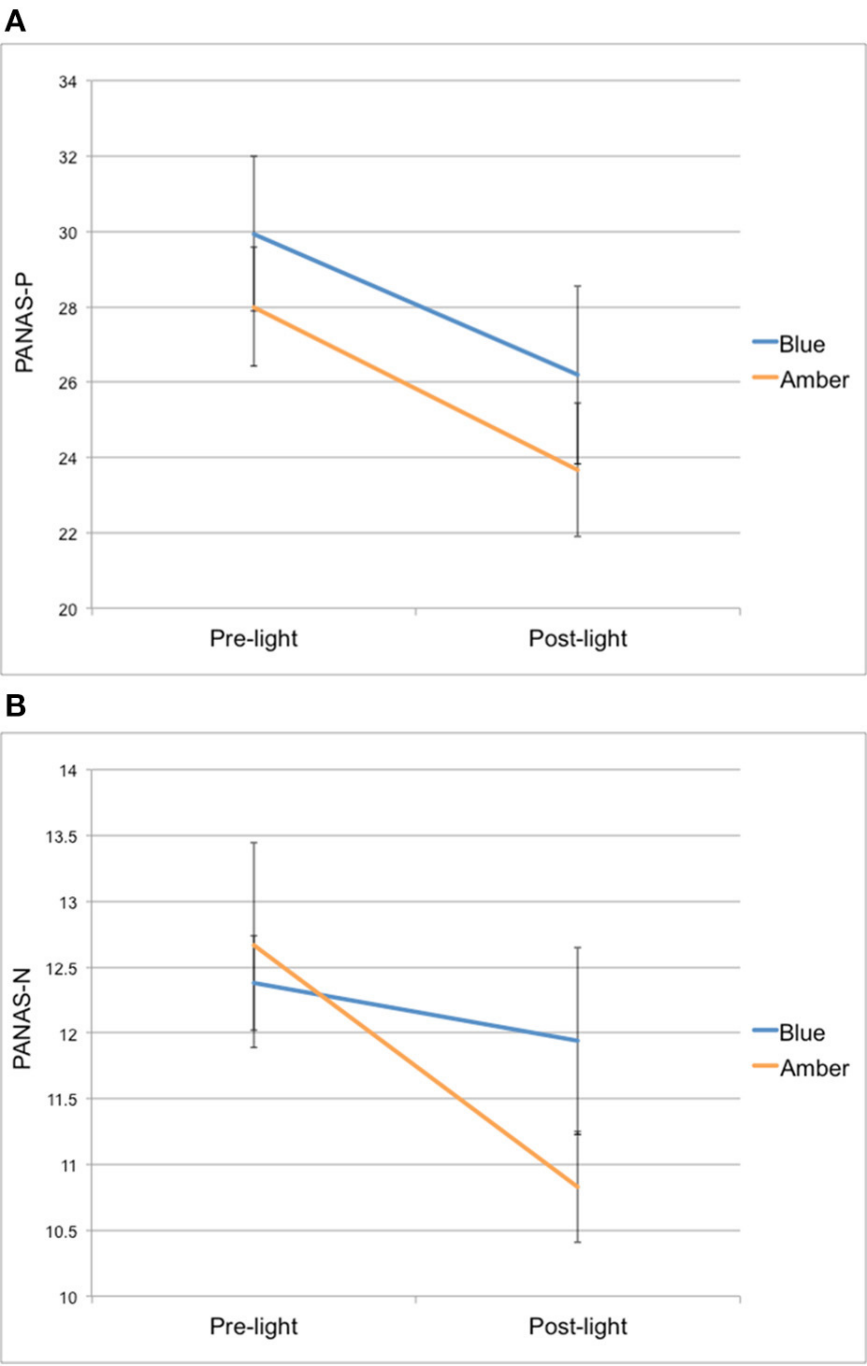
**(A)** PANAS positive affect (PANAS-P) and **(B)** negative affect (PANAS-N) scores from pre-light and post-light exposure for both groups.

All participants completed the study procedures at the same time of day to control for circadian effects. Specifics of the procedure are detailed in our previous paper (8). In brief, at 7:45 a.m., participants arrived for the study and completed informed consent as well as basic demographic questionnaires and cognitive tasks. Participants completed light exposure, cognitive tests, and mood assessments in a room located next to the magnetic resonance imaging (MRI) scanner. At ~8:30 a.m., participants completed the first PANAS. Participants also completed mood questionnaires, several cognitive assessments, such as general intelligence tests, and memory tests, and questionnaires about their daily habits. At 9:45 a.m., participants underwent a “blue light washout” period in an otherwise darkened room, with only two amber light devices placed on the table in front of them for 30 min. The light devices were adjusted until the pair of amber devices used during the initial washout period resulted in a 20-lux reading as measured by a light meter (Digital Lux Meter LX1330B) on each side of the participant's nose (i.e., at eye level). Participants were instructed not to look directly at the light devices, and to relax with their eyes open and maintain a generally forward gaze. This washout period was completed to ensure residual effects of outdoor and ambient lighting had dissipated before beginning the experimental light exposure manipulation while at the same time allowing participants to see their surroundings and continue engaging with the experimenter. At 10:15 a.m., the two Washout Period light devices were replaced with the *four* Exposure Period devices (see Figure 1B). Specifically, during the Exposure Period, participants were randomized to receive either 30 min of blue (*n* = 17) or amber (*n* = 12) light exposure. The 30-min Exposure Period was initiated by illuminating the two pairs of light devices (either blue or amber, depending on condition), with each pair mounted side by side on the desk in front of the participant, centered at the same location as the Washout Period amber lights at a distance of ~80 cm from the participant's eyes. During the 30-min Exposure and Washout Periods, participants maintained a forward gaze and completed two computerized practice tasks (a working memory and a multi-source interference task) to prepare them for their time in the scanner. To minimize blue light from the computer screen, an amber tinted plexiglass shield was placed in front of the laptop screen. Participants were asked to sit relatively still with their legs uncrossed and to maintain a forward gaze. Apart from completing the tasks on the computer using their fingers, participants did not move, stretch, stand up, drink, eat, or close their eyes during the light exposure sessions. Immediately after the light exposure period, at ~10:50 a.m., participants completed the second PANAS. At 11:00 a.m., participants were escorted to the MRI scanner, where the resting state scan was initiated at ~11:15 a.m. There are no measurements of the lux level inside the MRI scanner, but it was ~50 lux and the ambient light level was held constant across participants. The resting state scan was the first functional scan participants completed and was followed by longer versions of the working memory and multi-source interference tasks.

### Neuroimaging Methods

Neuroimaging scans were collected on Skyra 3T scanner (Siemens, Erlangen, Germany) using a 32-channel head coil. High resolution structural images were first acquired using a T1-weighted 3D MPRAGE sequence (TR/TE/flip angle = 2.1 s/2.33 ms/12°) over 176 sagittal slices (256 × 256) and a slice thickness of 1 mm (voxel size = 1 × 1 × 1 mm^3^). For the resting state functional scan, participants were instructed to let their mind wander while keeping their eyes open. Functional scans were acquired over 32 transverse slices (2.5 mm thickness; matrix: 88 × 84). Each volume was collected with an interleaved sequence (TR/TE/flip angle = 2 s/25 ms/90°). The voxel size of the T2^*^ sequence was 2.5 × 2.5 (i.e., with a 40% slice gap, allowing collection of 180 volumes within a 6-min acquisition time). The field of view (FOV) was 220 mm.

### Resting-State Pre-processing

Neuroimaging data were analyzed using the publicly available CONN functional connectivity toolbox (version 16.a; www.nitrc.org/projects/conn, RRID:SCR_009550), in conjunction with SPM12 (Wellcome Department of Cognitive Neurology, London, UK; http://www.fil.ion.ucl.ac.uk/spm). Raw functional images were realigned (motion corrected) and unwarped, slice-time corrected using the middle slice as the reference, and coregistered to each subject's high-resolution structural image in accordance with standard algorithms. The Artifact Detection Tool (ART; http://www.nitrc.org/projects/artifact_detect/) was used to regress out scans as nuisance covariates in the first-level analysis exceeding 3 SD in mean global signal intensity and scan-to-scan motion exceeding 0.5 mm. Six participants were excluded for an excessive number of outlier images (i.e., >36 scans flagged as outliers). These were included in addition to covariates for the six ridged-body parameters that characterize estimated subject motion, and used to regress out residual movement-related effects. Images were then normalized to Montreal Neurological Institute (MNI) coordinate space, spatially smoothed (8 mm full-width at half maximum), and resliced to a voxel size of 2 × 2 × 2 mm.

### Functional Connectivity Analysis

Using a standard seed-to-voxel approach, functional connectivity analyses were performed using the default functional connectivity processing pipeline in the CONN toolbox [for details, see (41)]. In this processing pipeline, physiological and other spurious sources of noise were estimated with the aCompCor method (42, 43) and subsequently removed together with the movement- and artifact-related covariates mentioned above. The residual blood oxygen level dependent (BOLD) time-series was then band-pass filtered (0.01–0.1 Hz). Every participant's structural image was segmented into gray matter, white matter, and cerebral spinal fluid using SPM12. Confounding effects of white matter and cerebral spinal fluid were removed through linear regression. Two seed regions of interest (ROIs) were placed corresponding to the left and right amygdala as defined by the Automated Anatomical Labeling (AAL) atlas (44). After the removal of confounds, the residual BOLD time-series from the bilateral amygdala seed ROIs were averaged to generate a mean time-series. Bivariate correlation maps (Fisher-transformed) were then computed with all other voxels in the brain to derive whole-brain connectivity maps. A group-level approach was used to compare differences in connectivity between the blue and amber groups controlling for the effects of age and sex. False positive control in seed to voxel analysis is implemented through a combination of a voxel height threshold (*p* < 0.001, two-sided; uncorrected) and a cluster level extent threshold (*p* < 0.05, cluster-size FDR-correction) (41).

### Directed Functional Connectivity Analysis: Granger Causality

In a second step, we investigated the strength of directionality of information flow [i.e., granger causality (GC)] between the amygdala and significant clusters from the functional connectivity analysis, using parametric GC (45, 46). Granger causality is a method for investigating causality between two time series, i.e., the extent to which one time series can predict the other. The strength of GC was estimated by quantifying the inter-relationships between their corresponding oscillatory mechanisms as a function of frequency (*f* ) of oscillations. For that, the raw time-series data were first band pass filtered using the Butterworth filter design with a higher cutoff frequency of 0.0028 Hz (*f*_1_) and a lower cutoff frequency of 0.1 Hz (*f*_2_). Next, the time-series for the bilateral amygdala and significant clusters from the functional connectivity analysis was zero-mean corrected in order to remove slow trends and physiological noise. Furthermore, the optimal model order for the parametric approach was calculated by comparing power spectra from the parametric and non-parametric approaches (45). Different model orders from 1 to 10 were tested, and the model order that yielded the lowest power difference was selected.

The threshold level for statistically significant GC strength, corrected for multiple comparisons, was estimated from surrogated data using permutation tests (45, 46) (*n* = 2,000) and a gamma function under a null hypothesis of no interdependence at the significance level of *p* = 0.0025.

## Results

### Affect Change From Pre- to Post-light Exposure

For PANAS-P scores, there was a significant main effect of time [*F*_(1, 26)_ = 25,67, *p* < 0.001], but no time × group interaction [*F*_(1, 26)_ = 0.13, *p* = 0.72] from pre- to post-light exposure. Overall, both light groups showed a decrease in their PANAS-P scores from pre- to post-light exposure (see Figure 1A).

For PANAS-N scores, there was also a significant main effect of time [*F*_(1, 26)_ = 6.15, *p* = 0.02], but no time × group interaction [*F*_(1, 26)_ = 2.32, *p* = 0.14] from pre- to post-light exposure. Overall, both light groups showed a decrease in their PANAS-N scores from pre- to post-light exposure (see Figure 1B).

### Strength of Functional Connectivity

**Figure 2:**
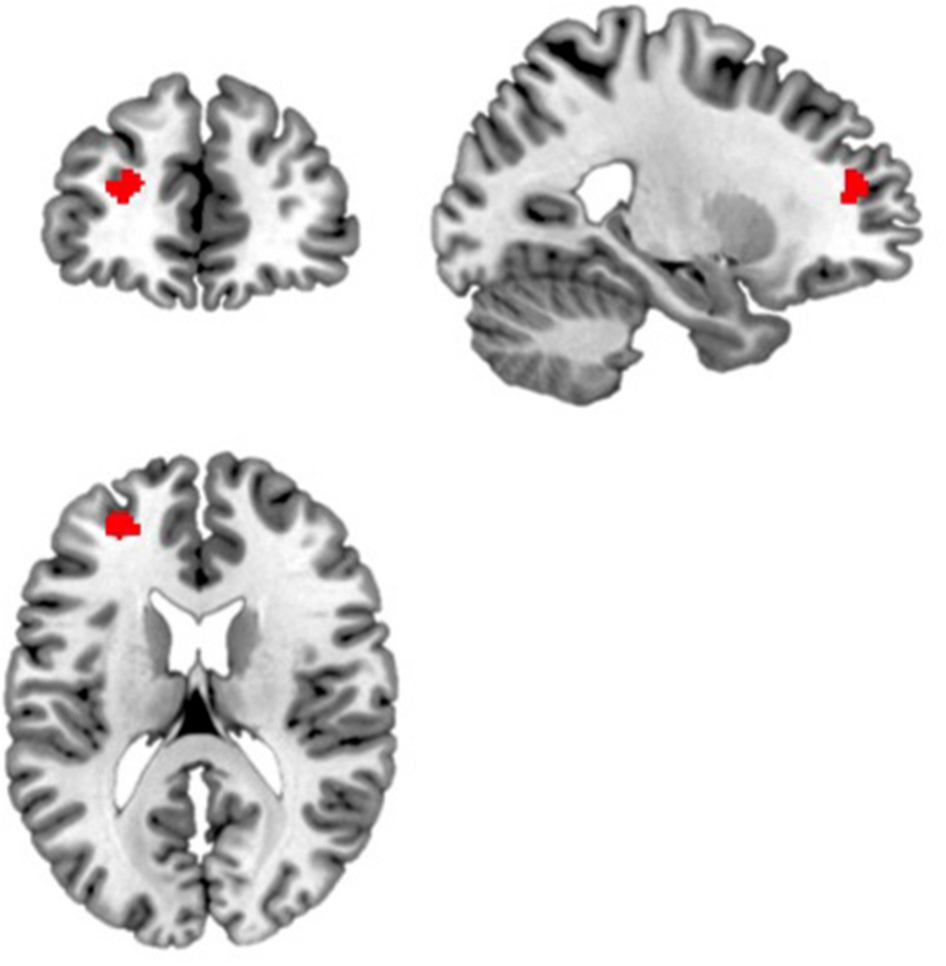
Blue light led to increased functional connectivity between the right amygdala and a region within the left dorsolateral prefrontal cortex. Displayed is the significant cluster of voxels (x = −24, y = 46, z = 18, k = 90, volume p-FDR corrected, *p* < 0.001) from the seed-to-voxel analysis using the right amygdala as the seed region.

Compared to participants in the amber light group, participants in the blue light group showed significantly greater positive intrinsic functional connectivity between the right amygdala and a cluster of voxels in the left dorsolateral prefrontal cortex (DLPFC) (x = −24, y = 46, z = 18, k = 90, volume p-FDR corrected, *p* < 0.001) (see Figure 2). No effect for the left amygdala was found. In summary, individuals who received blue light exposure showed significantly greater positive functional connectivity between the right amygdala and the left DLPFC compared to participants who received amber light.

### Strength of Granger Causality

**Figure 3:**
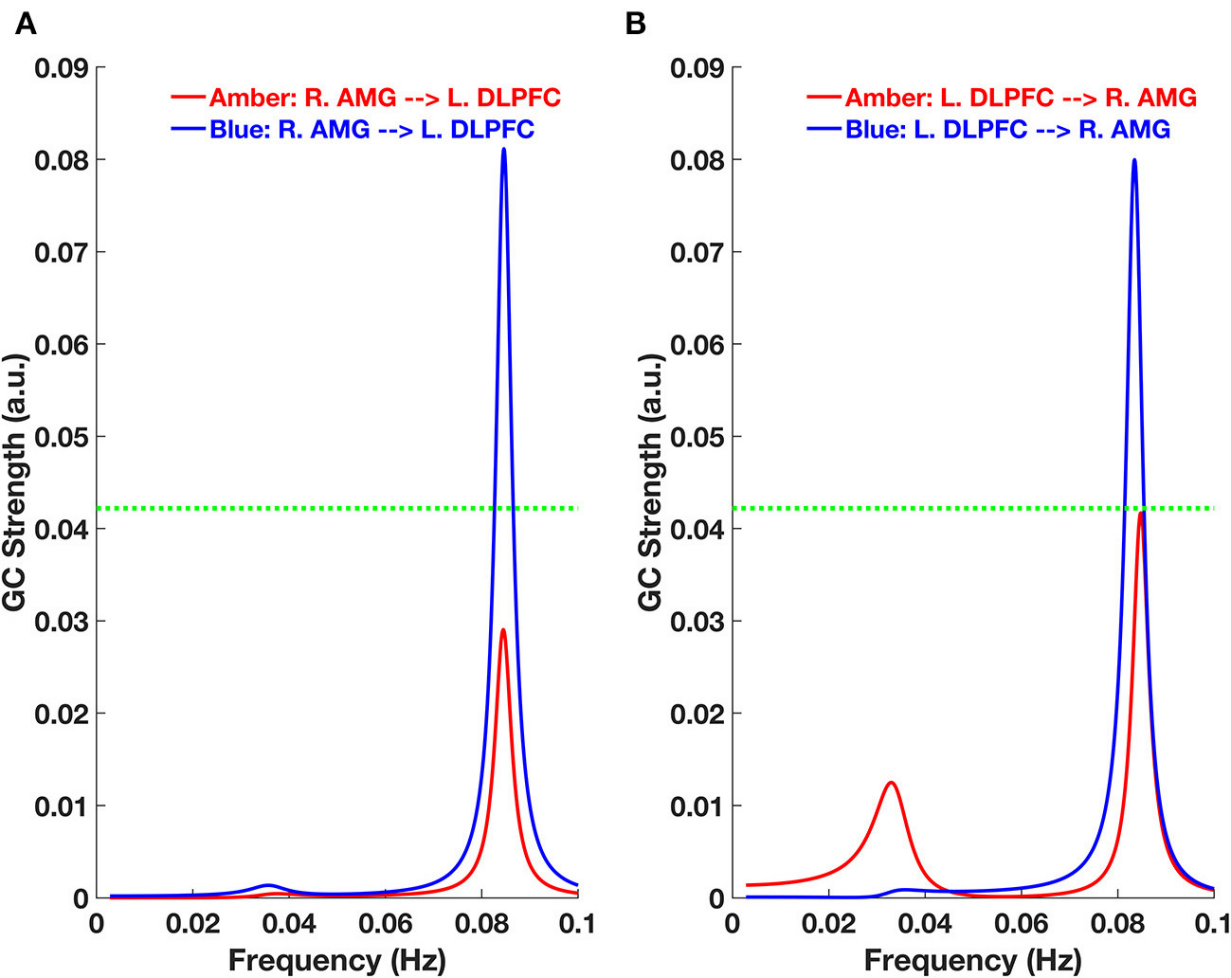
Granger Causality (GC)-frequency spectra for **(A)** feed-forward [right amygdala (R. AMG) to left DLPFC (L. DLPFC)] and **(B)** feed-backward (L. DLPFC to R. AMG) connections for the amber and blue light groups. The green dotted line here represents the threshold chosen for significant GC strength (~0.0422 at *p* < 0.0025, permutation test).

To determine the directionality of the connectivity between the right amygdala and left DLPFC, we employed GC and found that it was bidirectional. In other words, both the strength of the feed-forward [right amygdala (R. AMG) to left DLPFC (L. DLPFC)] (Figure 3A) and feed-backward (L. DLPFC to R. AMG) (Figure 3B) connectivity was significant for the blue-light group, but not the amber-light group. In Figure 3, the dotted line corresponds to a GC value of 0.0422, which represents a significance level at *p* < 0.0025 (*p* = 0.01/4, corrected for multiple comparisons).

### The Relationship Between Connectivity Patterns and Changes in Affect

**Figure 4:**
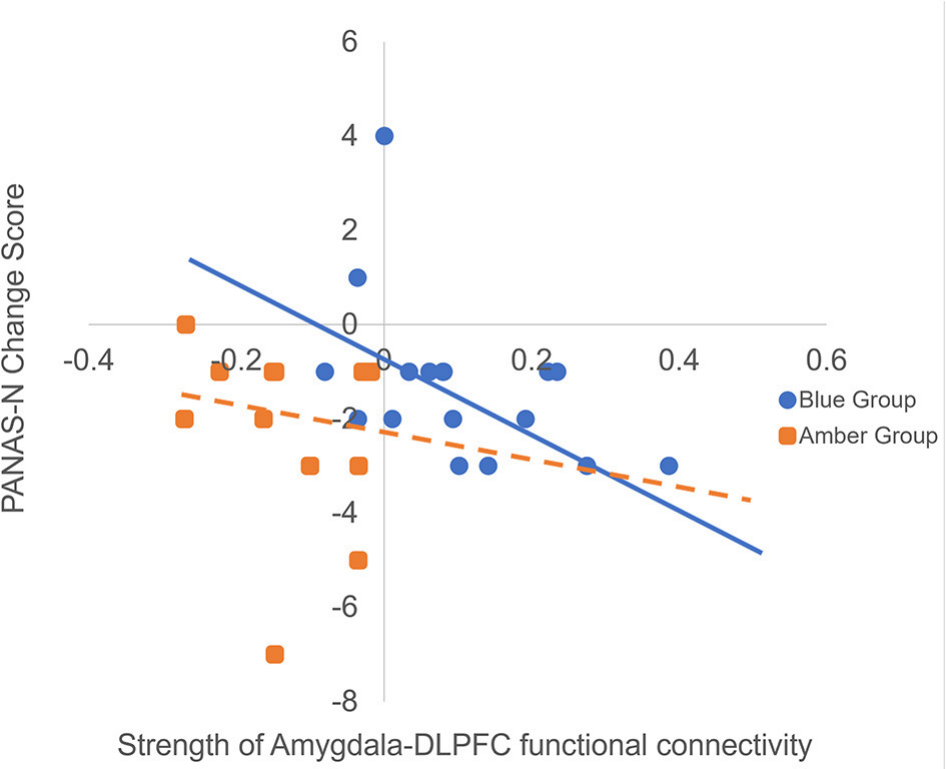
Here we are presenting the correlation between raw amygdala-DLPFC connectivity values and raw changes in PANAS negative affect (PANAS-N) scores from pre- to post-light exposure for the blue vs. amber groups (Greater negative values on the PANAS-N change scores indicate greater reduction in negative affect over time). We decided to present the raw scores rather than the Spearman rank order correlation for ease of interpretation, however we have included the trendlines for each group from the original analysis. The figure shows that increased functional connectivity between the amygdala and DLPFC was monotonically associated with reduced PANAS-N scores for the blue light group (ρ = −0.55, *p* = 0.03) but not the amber light group (ρ = −0.18, *p* = 0.55).

To investigate whether functional and directed connectivity was associated with changes in affect, we ran non-parametric Spearman's correlations between connectivity values and PANAS change scores. Spearman's correlations were used due to the small sample sizes and because the PANAS data violated assumptions of normality. For the blue light group, there was a statistically significant moderate negative relationship with PANAS-N change scores and functional connectivity (ρ = −0.55, *p* = 0.03), indicating that greater functional connectivity between the amygdala and DLPFC was monotonically associated with reduced negative mood following light exposure (see Figure 4). This relationship was not present for the amber light group (ρ = −0.18, *p* = 0.55). The strength of the monotonic relationship for directed connectivity was much smaller and non-significant for the blue light group (feed forward connectivity: ρ = −0.26, *p* = 0.33; feed backward connectivity ρ = −0.27, *p* = 0.29) and for the amber light group (feed forward connectivity: ρ = −0.24, *p* = 0.45; feed-backwards: ρ = −0.30, *p* = 0.34). There were no significant correlations between connectivity patterns and PANAS-P change scores.

### Potential Effects of Caffeine Consumption on Changes in Mood and Functional Connectivity

We compared the connectivity values for individuals who stated that they consumed caffeine prior to the assessments and those who stated that they did not consume caffeine (4 participants in each group consumed 1 caffeinated beverage prior to the assessment), and did not find a significant difference [total group: *t*_(27)_ = −1.30, *p* = 0.20; blue: *t*_(15)_ = −0.31, *p* = 0.76; amber: *t*_(10)_ = −2.03, *p* = 0.07]. There was also no difference between individuals who drank caffeine and those who did not on PANAS-N or PANAS-P change scores [PANAS-N: total group: *t*_(27)_ = 0.33, *p* = 0.74; blue: *t*_(15)_ = −0.26, *p* = 0.80; amber: *t*_(10)_ = 0.91, *p* = 0.38; PANAS-P: total group: *t*_(27)_ = −0.83, *p* = 0.42; blue: *t*_(15)_ = 0.33, *p* = 0.75; amber: *t*_(10)_ = −1.50, *p* = 0.17].

## Discussion

This study investigated the effects of exposure to 30-min of blue wavelength light on subsequent functional brain connectivity at rest and associations with changes in affect. The results showed that individuals exposed to a single 30-min pulse of blue wavelength light showed greater positive functional connectivity between the right amygdala and the left DLPFC than individuals exposed to an equivalent period of amber light, and that greater functional connectivity between these two areas was associated with greater decreases in negative mood. These findings build upon results from previous studies that have demonstrated that even after 50 s of blue light vs. green light exposure, there are transient increases in activation within the right amygdala (31). In line with this, there is evidence to suggest that the amygdala receives direct projections from ipRGCs (29) and that swift modulations of amygdala activation may contribute to the antidepressant effect of blue wavelength light (47). Specifically, previous work also showed enhanced functional connectivity between the left amygdala, the voice area of the temporal cortex and the hypothalamus during an auditory emotional task in the context of blue vs. green light exposure (32). The authors proposed that their results suggest that blue light exposure leads to increased limbic reactivity to emotional stimuli which may lead to quicker behavioral adaptations to emotional challenges, which could ultimately enhance emotional regulation. Our results support those previous findings that it is likely that blue wavelength light exposure leads to immediate amygdala activation and associated increases in arousal via the direct amygdalar projections from the ipRGCs. Our study also builds upon previous functional connectivity findings by showing that blue light exposure was associated with greater bi-directional information flow between the amygdala and DLPFC. We speculate that in order to respond to these increases in arousal, the DLPFC may, in turn, respond by engaging in cognitive control strategies, such as selectively directing attention to stimuli in the environment, recruiting emotion regulation strategies by modulating amygdala activation, and influencing social decision-making. Our interpretation is similar to Vandewalle et al.'s (32) interpretation of their functional connectivity findings and may explain why blue wavelength light exposure is an effective treatment method for individuals with seasonal and non-seasonal depression (10–13), but future research will be necessary to explore these possibilities further.

A growing body of work demonstrates that depression is characterized by impaired cognitive and emotional processing which may reflect increased activation within areas such as the amygdala and decreased activity within areas involved in the effortful regulation of emotional behavior, such as the DLPFC and other frontal regions (48). A number of studies demonstrate that during active emotion regulation, individuals with depression (compared to healthy control subjects) show less negative (i.e., inverse or anti-correlated) connectivity between the amygdala and the PFC and that this reverses when symptom severity decreases to levels no longer considered clinically significant (49). Functional resting-state studies, however, suggest that individuals with depression show reduced positive connectivity between the amygdala and several prefrontal regions, in comparison with healthy controls (50–52). It should be noted that the majority of these studies have not reported a direct link between the *right* amygdala and the *left* DLPFC, specifically. One study, however, showed that after successful transcutaneous vagus nerve stimulation (tVNS) individuals with depression showed increased resting state functional connectivity between the right amygdala and the left DLPFC which were associated with decreased depression symptoms (53). The results from the present study suggest that blue light exposure may have an immediate beneficial effect on functional connectivity between the amygdala and PFC similar to that of tVNS by increasing information flow between these regions at rest. This is further supported by our finding that greater amygdala–DLPFC connectivity following blue light exposure was associated with greater decreases in negative affect from pre- to post-light exposure, just as tVNS was associated with decreases in depression scores. The expression of depression can vary greatly between individuals and the circuits that may be impacted by depressed mood are equally manifold and can include dysregulated reward processing, heightened reactivity to negative environmental cues, increased default mode activity, and more (54). Different treatments for depression can therefore influence depressive symptoms in various ways by targeting distinct neuronal circuits. It is possible that blue wavelength light may have a unique impact on modulating amygdala and DLPFC reactivity, similar to tVNS, which may be particularly useful for certain individuals, but future research will be necessary to replicate and expand on these findings. In addition, future research should consider that light exposure may be even more beneficial when used in conjunction with emotional learning/therapy. For example, we speculate that blue light exposure while practicing emotional regulation skills might have an even stronger effect than blue light exposure without affective content. As previous studies have proposed that exposure to blue light may facilitate learning through increased norepinephrine release from the brainstem (9), using light therapy in conjunction with therapeutic approaches that enable better emotion regulation abilities, such as cognitive reappraisal, should be the focus of future research. Some evidence already suggests that such interventions may be effective, as individuals who underwent exposure therapy for panic and post-traumatic stress disorder showed greater reduction in anxiety and depression scores if the therapy was accompanied by concurrent bright light (vs. dim light) exposure (55). Another study showed that bright light exposure during fear extinction learning in healthy individuals suppressed fear acquisition and enhanced fear extinction (56).

It is important to highlight, that while we interpret our results as being the result of blue wavelength light exposure, it is also possible that amber wavelength light had a unique effect on dampening the strength of amygdala—DLPFC connectivity (e.g., amber light could have a “relaxing effect”) rather than that blue light exposure led to an increase in connectivity between these two areas, although the potential mechanism for such an effect is unclear. The cross-sectional design also leaves the possibility open that a third variable not under study investigation contributed to the findings, such as that individuals in the blue light group had stronger amygdala-PFC connectivity at baseline and that the observed correlations with changes in mood are explained by baseline differences in brain function.

It should be noted that participants in our sample were free from known psychological disorders, making the detection of subtle changes in mood/affect more challenging. The fact that we did observe a moderate to strong association between the strength of functional connectivity and changes in negative affect (ρ = 0.55) makes these results notable. However, replication of this study in a clinical sample of depressed individuals would shed additional light onto the immediate effects of blue light on mood and allow extension to of these findings to clinical depression. While our stimulation period lasted only 30 min on a single occasion, it is conceivable that exposure to blue wavelength light for longer durations, perhaps over several weeks, may positively impact the coupling of the amygdala-DLPFC connection for individuals with clinical depression, which in turn may improve effective emotion regulation skills and executive function. There is some evidence for this, as Fisher et al. (33) demonstrated that 3 weeks of bright light therapy in healthy males reduced bilateral amygdala and prefrontal reactivity to threat (i.e., angry and fearful faces) in a dose-dependent manner; and that left amygdala and prefrontal connectivity also increased in a dose-dependent manner (33). However, these changes did not correspond with changes in mood or stress.

As participants in this study were not clinically depressed, it is unclear how these neurobiological changes would correspond to changes in mood in a sample of individuals with clinical psychopathology. Further research is necessary to understand better how bright light, particularly in the blue wavelengths, affects functional brain responses in individuals with depression and how those may be associated with improved mood.

Of note, the effects of blue wavelength light on amygdala-DLPFC functional connectivity at rest may represent just one mechanism by which light affects emotion and cognition. The ipRGCs, which are believed to be the primary system by which blue light affects brain activation and circadian physiology, also project to other brain areas important in mood disorders, such as the habenula and hypothalamus. While we focused on the amygdala and prefrontal cortex here, these other mood-relevant brain structures may also be influenced by blue light exposure (57–60). Future studies should therefore expand their analyses to other brain areas that may contribute to some of the mood enhancing effects of blue wavelength light. Other lines of work have focused on the effects of light exposure on the influence of serotonin signaling, which may interact with the effects of blue light on cortico-limbic reactivity. For example, individuals with seasonal affective disorder (SAD) display enhanced serotonin transporter signaling during the winter months, which normalizes with successful treatment or during natural summer remission (61). Another study showed that after a bright-light intervention in healthy males, differences in the 5-HTTLPR genotype moderated changes in functional brain responses between the amygdala and PFC toward threat (33). Future research would likely benefit from investigating the potential interplay between serotonin signaling and amygdala-PFC connectivity on emotional functioning and mood after blue light therapy.

It should be pointed out that our results did not support the notion that a single exposure to blue wavelength light during the day would lead to increased positive and decreased negative affect. Both groups showed a decrease in the *intensity* of emotion for positive and negative affect over time, raising the possibility that the effects of increased amygdala-PFC connectivity following blue light exposure resulted in a global moderating effect on mood. In other words, it is possible that blue light exposure may simply shift mood away from the extremes and toward the middle ranges of intensity. Of course, it is also possible that group differences in mood changes were not detected due to other confounding study effects, such as intense positive and negative feelings at the beginning of the study, which may have dissipated toward the end for both groups (e.g., feeling excited, but apprehensive at the beginning of the day because of the novelty of being in a research study and/or the MRI acquisition). Future research would benefit from investigating the immediate changes in affect in response to light exposure in greater detail, including using more precise forms of self-report measures, investigating differences between healthy controls and those with depression, as well as the potential effects of time of day of exposure.

The results of this study should be interpreted with several limitations in mind. This study employed a between-subjects design and it is therefore possible that the observed results could be explained by individual differences between the groups rather than the light condition. Additionally, the sample sizes of the two groups were relatively small and not equivalent in size across groups which means that the larger sample in the blue light group could have yielded a different signal to noise ratio than in the amber light group. It is also important to mention that our results were considered as statistically significant using a cluster-level threshold. Eklund et al. (62) have raised the possibility that this can potentially result in inflated false-positive rates which should be kept in mind when interpreting our findings. This study also relied on participants' self-report regarding their sleep-wake history rather than more objective measurements, such as actigraphy data, potentially affecting brain responses to light. Additionally, while we attempted to control for circadian phase effects by performing the experiment at the same clock time for each participant, control would have been enhanced by regulating and monitoring their sleep in the sleep lab to control for circadian effects. These methodological limitations may have influenced the results of this study and it should therefore be replicated using a within-subjects design with a larger sample size. Further, in this study, we aimed to investigate one potential mechanism through which blue wavelength light might influence affect and chose to study a group of healthy individuals without psychopathology. However, it is unclear how these results apply to individuals with clinically significant levels of depression. For example, it is possible that blue light exposure has a different effect on depressed individuals, and a similar study incorporating a sample of currently depressed individuals is necessary to help elucidate this. In addition, to better understand how light therapy may alter brain function in depressed individuals, subsequent research would benefit from investigating changes in brain function at rest, as well as in response to emotional tasks, before and after light therapy. Finally, while this research contributes to our understanding of the mechanism by which blue wavelength light leads to improved mood, additional research is necessary to determine the precise pathways through which light influences functional brain responses and how those immediate modulations in functional connections lead to long-term behavioral and emotional changes in mood and well-being.

## Conclusion

The present findings suggest that a single 30-min exposure to blue wavelength light during the day increased the bi-directional strength of positive functional connectivity between the right amygdala and the left dorsolateral prefrontal cortex and that this was associated with improvements in affect, particularly a reduction in negative affect. These findings suggest a neurobiological mechanism that may underlie the often-observed association between light exposure and improved mood.

## Data Availability Statement

The raw data supporting the conclusions of this article will be made available by the authors, without undue reservation upon request.

## Ethics Statement

The studies involving human participants were reviewed and approved by University of Arizona IRB. The patients/participants provided their written informed consent to participate in this study.

## Author Contributions

AA contributed to the study design, data collection, data analysis, and manuscript preparation. ND and SB contributed to the data analysis and manuscript preparation. AR and JV contributed to the manuscript preparation. WK contributed to the study design, data analysis, and manuscript preparation. All authors contributed to the article and approved the submitted version.

## Conflict of Interest

The authors declare that the research was conducted in the absence of any commercial or financial relationships that could be construed as a potential conflict of interest.
